# We are treated differently: Experiences of men who have sex with men in South African clinics

**DOI:** 10.4102/safp.v67i1.6050

**Published:** 2025-02-20

**Authors:** Tshivhase T. Ravele, Raikane J. Seretlo, Mathildah M. Mokgatle

**Affiliations:** 1 Department of Public Health, School of Healthcare Sciences, Sefako Makgatho Health Sciences University, Tshwane, South Africa

**Keywords:** lived experiences, men who have sex with men, public and private clinics, healthcare facilities, healthcare services, healthcare workers.

## Abstract

**Background:**

Men who have sex with men (MSM) are regarded as a key population that needs specialised healthcare services to address their healthcare needs. This is because of their sexual practices. Men who have sex with men encounter positive and negative experiences when accessing healthcare services in public health care facilities. This study explored and described the experiences of MSM about accessing healthcare services in public healthcare facilities. The study was conducted in Ekurhuleni district, situated in the East of Gauteng province, South Africa.

**Methods:**

A descriptive, explorative, qualitative approach was employed to investigate the experiences of MSM about accessing healthcare services. In-depth interviews were conducted with 17 MSM aged 18 years and above.

**Results:**

Overall, MSM had both negative and positive experiences when accessing healthcare services. However, mainly the negative in the public healthcare facilities, whereas positive were experienced in the private healthcare facilities.

**Conclusion:**

The negative experiences of MSM, the judgemental and discriminatory attitudes of healthcare workers towards them and the scarcity or unavailability of resources and healthcare services to address their specific healthcare needs in healthcare facilities have created interpersonal and structural barriers, which prevent MSM from accessing healthcare services.

**Contribution:**

These findings suggest the need for the training of healthcare workers to be truly caring, the allocation of resources and healthcare services to addressing MSM-specific healthcare needs in healthcare facilities and the creation of an accommodating environment for them.

## Introduction

Healthcare facilities are expected to provide quality, affordable and equitable healthcare services, which include behavioural and social services.^1^ In addition, according to the Robert Wood Johnson Foundation Initiative on the Future of Nursing^2^ healthcare facilities should ensure social justice by enhancing equal healthcare services to be rendered regardless of one’s personal characteristics background, race and sexual orientation. Accessible health services are those that are available to everyone who requires them and are provided without discrimination, regardless of where they were born, what language they speak, their cultural or religious background, their ability, sex or gender.^3^ The World Health Organization’s (WHO).^4^ facts sheet on quality health services highlighted that the degree of quality care is to evaluate, which health services for individuals and populations increase the likelihood of desired health outcomes.

Men who have sex with men (MSM) are referred as a distinct group of men, inclusive to men who self-identify themselves as gay, bisexual or transgender; detained self-identified heterosexual men who, owing to their situations, have intentional sexual relations with other men.^5^ Pachauri et al.^6^ stated that MSM include individuals who identify as gay, bisexual or straight, as well as those who do not name a sexual preference at all. It seems to still be a challenge for MSM to receive a comprehensive, equal and quality healthcare services. For examples, several studies have shown that MSM continue to experience prejudice, discrimination, judgement and being stigmatised in the healthcare facilities.^7,8,9^

Some of the challenges experienced in the healthcare facilities are lack of supportive structural resources, inadequate healthcare providers training and knowledge related to MSM health matters and a lack of healthcare coordination.^10,11,12^ Most of the above-mentioned challenges seem to be occurring across the world, that is, globally, studies have shown that MSM reported and experienced stigma by the healthcare system, lacked MSM-friendly services and insufficient resources.^13,14^ Again, in some of the sub-Saharan Africa (SSA) countries MSM continue to experience stigmatisation and feel healthcare facilities are unsafe.^10,15^ In addition, MSM end up not disclosing their sexual orientations to healthcare providers because of a lack of confidentiality, fear of stigma and discrimination, shame and mistreatment at the health facilities, and fear of the healthcare worker’s reaction after they disclose their sexual orientation to them.^16^

In South Africa, there are several policies and interventions introduced by the government to ensure that all individuals including MSM are included in the healthcare services. The examples of these policies are the Constitution of the Republic of South Africa, The South African National lesbian, gay, bisexual, transgender, and intersex (LGBTI) human immunodeficiency viruses (HIV) Plan (2017–2022), The HIV and/or acquired immunodeficiency syndrome (AIDS) and sexual transmitted illness (STI) National Strategic Plan (NSP) (2007–2011), and National Health Insurance (NHI). However, MSM within the country still continue to experience healthcare challenges such as scarcity of healthcare services for preventing and treating health conditions such as HIV.^17^ Furthermore, other South African studies found that MSM experience stigma and lack of relevant knowledge, skills and training to manage the particular health needs and vulnerabilities by healthcare providers.^18, 19^

If the aforementioned challenges are not addressed, MSM individuals will continue to experience some healthcare challenges such as mental healthcare issues like depression, stress, anxiety.^20,21,22^ Other healthcare issues that might increase among MSM could be the spread of STIs such as HIV, gonorrhoea, Chlamydia trichomoniasis.^23,24,25,26,27^ In addition, some social challenges such as higher rates of alcohol and drug intake might result.^28,29^ Men who have sex with men will continue to delay and avoid seeking healthcare services, blaming it on healthcare facilities.^30^

Little is known about how MSM differentiate healthcare services received in public and private clinics; hence, our study aimed at exploring and describing the experiences of MSM in accessing healthcare services in Ekurhuleni district, Gauteng province, South Africa. The study’s findings will contribute to the body of knowledge and influence policymakers to develop sensitised policies that would enhance quality and safe healthcare services for MSM. Thus, incorporating healthcare services from both public and private sectors and enhancement of the newly introduced NHI within the country.

## Research methods and design

### Study design

We conducted a descriptive-explorative qualitative study with the intention of understanding the experiences of MSM when accessing healthcare services in public healthcare facilities.

### Study setting

The study was conducted in Ekurhuleni district, situated in the East of Gauteng province, South Africa. Ekurhuleni district is divided into three service delivery regions (SDRs), Southern service delivery region (SSDR), Eastern service delivery region (ESDR) and Northern services delivery region (NSDR). Ekurhuleni health district consists of 93 fixed primary health care (PHC) facilities, 86 mobile points (of which 32 are located in informal settlements and 54 in formal settlements) and 6 district hospitals. This study was undertaken in the SSDR.

### Population and sampling strategy

The participants of this study were MSM individuals. Our study research question was ‘What are the experiences of MSM in accessing health care services in Ekurhuleni District, Gauteng province?’. A total of 17 MSM participants were sampled using snowballing method because of the small size, because of the difficulty in finding MSM individuals as the result of the complexity and sensitive nature of the researched phenomenon. The principal investigator commenced with presenting the study purpose to local LGBTI committee to which most MSM were affiliated and asked for the identification of prospective participants. The principal investigator applied snowballing method by approaching one open MSM participant. The first participant was recruited through a one-on-one private conversation in which all the details of the intended study were explained to the prospective participant.

### Recruitment of the participants

The recruitment of the participants in the study was carried out immediately after obtaining ethical clearance from Sefako Makgatho Health Sciences University Research and Ethics Committee. An appointment with the first participant was made telephonically, in which the prospective participant agreed to meet the principal investigator. After a successful interview with each participant, the principal investigator would ask him to identify another potential participant who also met the criteria to participate in the study.

Continually, every MSM individual who participated was asked to refer other MSM individuals; at the end, all MSM acted as informants and assisted with identifying other members for inclusion in the sample until the 15th participant, where data were saturated. The principal investigator continued to recruit until the 17th participant to confirm data saturation. This means that the principal investigator stopped with the recruitment of MSM individuals at the point where no new information from MSM participant’s responses was emerging.

### Data collection

Data were collected through one-on-one interviews with the participants in this study. The interviews were used as the most appropriate method for obtaining detailed information regarding the experiences of MSM about accessing healthcare services in healthcare facilities. Initially, interviews with the study participants were to be conducted at local clinic in a private room or any other unoccupied consulting room available on the day of the interview. It turned out that all the participants could not make it to the selected venue because of their busy schedules and the distance from their places of residence. The principal investigator and the participants agreed to meet at a neutral venue for the purpose of conducting the interviews. Some of the interviews were conducted in the principal investigator’s vehicle to ensure privacy and avoid external disturbances. All 17 interviews were conducted by the principal investigator using a digital recorder, later transcribed. The principal investigator was guided by a semi-structured interview guide, which had open-ended and probing questions. Each interview lasted about 45 min to an hour.

### Data analysis

The principal investigator applied and followed the steps of thematic analysis method developed by Braun and Clarke.^31^ In short, the principal investigator immersed himself in the data to become familiar with the data through repetitive reading, generated initial codes, then transferred all the transcripts into NVivo 12 software to develop themes, and reviewed and compared the themes (Box 1). Furthermore, the principal investigator wrote the interpretive perspective relating it to the data extracts to convey to the reader a complete, informative interpretation of the data in the study.

BOX 1 A summary of emerged themes.

**Table:** 

**Themes** Experiences in the public healthcare facilities Experiences in private healthcare facilities Mislabelling MSM during consultation in the public health facilities Judgemental attitudes towards MSM in public health facilities MSM discriminated and stigmatised in public healthcare facilities

MSM, Men who have sex with men.

### Ethical considerations

Ethical approval to conduct this study was obtained from Sefako Makgatho Health Sciences University and Sefako Makgatho Health Sciences University Research and Ethics Committee (No. SMUREC/H/219/2017:PG). The principal investigator explained participants’ right to withdraw at any stage without negative consequences. The principal investigator also guaranteed willing participants anonymity, privacy and confidentiality throughout the interview process and the entire study. In addition, these were achieved by using pseudo-names for all MSM. All MSM individuals who were willing to participate were required to give consent to participate prior the commencement of an interview, through the signing of a written consent form.

## Results

### Sample characteristics

The mean age of the participants was 28 years. Only 1 of the 17 MSM had attended school up to matric level, 3 had attended school up to a secondary level, and the remaining 13 had received tertiary education. Eleven participants had no medical aid cover, meaning that 6 had medical aid cover. Thirteen of the participants were in a same-sex sexual relationship, while the remaining 4 were not in a sexual relationship at all. A total of 6 participants were dating 1 sexual partner, 3 were dating 2 sexual partners, 1 was dating 3 sexual partners, 1 was dating 4 sexual partners, 1 was dating 5 sexual partners, and the last participant was dating countless sexual partners (Table 1).

**TABLE 1:** Sociodemographic characteristics men who have sex with men (*N* = 17).

Characteristics	MSM frequency	Mean	s.d.
**Age (years)**	-	28.12	5.79
21–25	7	-	-
26–30	5	-	-
31–35	4	-	-
36–40	0	-	-
41+	1	-	-
**Highest qualification**	-	-	-
Secondary	1	-	-
Matric	3	-	-
Tertiary	13	-	-
**Have medical aid**	-	-	-
Yes	6	-	-
No	11	-	-
**Currently in a sexual relationship**	-	-	-
Yes	13	-	-
No	4	-	-
**Type of relationship**	-	-	-
Same-sex	13	-	-
N/A	4	-	-
**Number of sexual partners**	-	-	-
0 Partner	4	-	-
1 Partner	6	-	-
2 Partner	3	-	-
3 Partner	1	-	-
4 Partner	1	-	-
5 Partner	1	-	-
Countless partner	1	-	-

MSM, Men who have sex with men; N/A, not applicable; s.d., standard deviation.

### Experiences in the public healthcare facilities

The majority of the participants reported that they encountered inadequate treatment from the healthcare workers in the public healthcare facilities. Other participants highlighted that the healthcare workers were uncomfortable in assisting them, as they were surprised to find out about their sexuality, healthcare workers laughed at them, were rude and they were not giving them full attention during their visit to the healthcare facilities. In addition, some participants stated that healthcare workers were unable to render their specific health issues related to sexual health but offered them basic care such as painkillers instead of proper referral:

‘Sometimes they can’t help you with the problem that you are having. You know those things like, what can I say. You were having sex and your anal was torn, they can’t help you, they just give you pain blocks … Go home and drink your pain blocks then you will be fine. You know sometimes I don’t think they see the use of that because they will tell you that even if we examine you, we cannot help you with anything because we are going to send you at the hospital or give you pain blocks.’ (Gift, a 23-year-old bottom gay)

‘Not really happy with the treatment. Most of the time when you present or go with your partners, the health care workers will kind of like go out to get other health care workers inside the room and you end up not having privacy … they end up discussing your issues and stuff. When I visit the clinic, they just say you can buy yourself medication because we don’t have that kind of medication.’ (Ishmael, a 31-year-old bottom gay)

Public healthcare workers are seen to be uncomfortable rendering services to the MSM, some were found to leave MSM in the consultation room and some blaming MSM for having anal sex. Men who have sex with men participants complained about the lack of privacy being afforded during consultation.

### Experiences in private healthcare facilities

Private healthcare facilities seemed to be reported as a good space for seeking healthcare help as MSM. Some participants reported that they had good interpersonal relations with the healthcare workers. They reported that the healthcare workers could share jokes with them without making them feel offended. The participants further reported that the reactions of the healthcare workers towards them were good during their visits to the healthcare facilities:

‘It’s actually easy to interact with health care workers at Private Clinic 1, because most of the guys there are actually trained to work with homosexuals. Most of the staff from your receptionist, even if you fall sick the moment you get there, collect your file, the clerk just makes you feel better. She is always bubbly just because it’s a men’s clinic. There are women who work there but the way they work it shows that they were trained to work with MSM.’ (Fezile, a 25-year-old bottom gay)

‘In the private doctors, things are better because they give you attention. They listen to you when you talk to them.’ (Rods, a 25-year-old top gay)

The MSM believed that in the private healthcare facilities the staff members are well-trained about caring and managing homosexual people. Men who have sex with men reported that every staff member came across bubbly and friendly, they pay attention to what MSM patients are there for and they feel treated like human beings.

### Mislabelling men who have sex with men during consultation in the public health facilities

Most of the participants indicated that they had experienced incidents where healthcare workers name shame them and force them that they belong into LGBTI category as gay people. In addition, participants outlined that healthcare workers call them with different titles such as Mrs. lady, mam, girl, while some were addressed by their original names when accessing healthcare services in healthcare facilities by healthcare workers:

‘I get those who says Mrs. and those who says Mr.’ (Thando, a 25-year-old bottom gay)

‘They were calling me lady, mam, you know … girl …’ (Thabang, a 25-year-old bottom gay)

‘They just call me with my straight name, Freddy. That’s it! They don’t call me he or she, they call me by my straight name just because they did ask if they supposed to call me, she or he.’ (Freddy, a 31-year-old bottom gay)

### Judgemental attitudes towards men who have sex with men in public health facilities

Some of the negative experiences encountered by participants when accessing healthcare services in public healthcare facilities are the judgemental attitudes of healthcare workers towards MSM. Some participants felt that homosexual people are not welcome in public healthcare facilities and some reported that healthcare workers bring the issue of religion into their sexuality, which was portrayed as something that God does not allow:

‘Honestly, firstly it was uncomfortable and secondly it was actually not nice. it was not nice because at that time the staff members were very judgmental with my sexuality and age. They were very judgmental, so leading to events whereby in most cases I wouldn’t go to clinics, let me just sit home and maybe I will get better. To an extent of being scared to tell my parents that I’m sick. Those things. So, I decided, I told myself I’m no longer going to use community clinics, never. What I’m concerned with is as soon as I tell them what they are up to how handle the situation, that’s the problem actually. Some are friendly and the boring part is why did they gossip about the patient?’ (Fezile, a 25-year-old bottom gay)

### Men who have sex with men discriminated and stigmatised in public healthcare facilities

Some participants described acts of discrimination against them by healthcare workers when they accessed healthcare services in public healthcare facilities. Furthermore, participants indicated that healthcare workers stated that only heterosexual men have lesser risk of contracting sexual illness than MSM and other health care workers did not provide a fair chance for MSM to make suggestions about the healthcare services received indicating that it is not within their patients’ rights:

‘Also, with STIs, circumcision, they didn’t mention gay people, I am telling you. One thing they mentioned was that you know when you circumcised and sleeping with your woman it’s much easier and it’s 60% less chance that you contract HIV and use a condom when you sleep with your woman. That is the only time, and I find it funny that we can leave here and find somewhere around the hospital. They only accommodate, even their posters are about straight sex. They would say come and test and also sex. It’s very weird that there is no gay guy there mentioned, men to men, not unless that they see you, they will basically say “OK, come also, Choma,” things like that. Why don’t they say that in this platform we accommodate men to men, and this is heterosexual? We feel like our government also is pressing us down, as much as we feel we are free, but honestly when it comes to health sector, we still have a long way to go.’ (Bafana, 29-year-old versatile gay)

‘Because they told us it’s not within our rights to make suggestions in this clinic because we are not normal human beings.’ (Leyla, a 21-year-old bottom gay)

## Discussion

The aim of our study was to explore and describe the experiences of MSM about accessing healthcare services in public healthcare facilities. The majority of participants reported encountering inadequate treatment from healthcare workers in public facilities. In addition, some participants highlighted that healthcare workers were ill-equipped to address their specific sexual health needs, often providing only basic care, such as painkillers, instead of appropriate referrals. This evidence supports previous findings that showed MSM respondents were not satisfied with the services rendered to them when accessing healthcare services and indicated that some services that they required were not available such as STIs related health services.^32^ Findings from earlier studies^33^ have shown that healthcare facilities lack MSM-friendly HIV-related health services.

Men who have sex with men from our study noticed that healthcare workers were often uncomfortable, displaying surprise upon learning of the participants’ sexuality. This discomfort manifested in inappropriate behaviour, including laughter, rudeness and a lack of attention during consultations. Public healthcare workers were also observed to be uneasy when rendering services to MSM, with some even leaving the consultation room or blaming MSM for engaging in anal sex. The main healthcare climate factors that affected prevention were that MSM were not free to be themselves, MSM were not understood by healthcare providers and that MSM did not feel that healthcare providers cared about them.^34^ Furthermore, an earlier study^35^ found that fewer MSM highlighted being refused health services because of their MSM status. These findings corroborate the findings of another study^36^ where LGBT patients failed to disclose their sexual orientation because of their experiences of past discrimination or the expectation of negative reactions from healthcare providers. In addition, a study that was conducted among nurses showed that some nurses used their cultural and religious beliefs as the reasons why they were not comfortable to assist and being ready to talk about MSM-related health issues.^12^ Again, our study corroborate the findings of previous investigations that revealed MSM noticed some judgemental and discriminative communication ways from the healthcare providers such as the tone of their voice, both verbal and non-verbal cues.^37^ Men who have sex with men raised privacy concerns and complained about the lack of confidentiality during consultations. Similarly, our findings align with another study,^33^ which found that MSM clients complained about healthcare facilities not having confidentiality and privacy.

In contrast, private healthcare facilities were generally reported as more welcoming and accommodating spaces for MSM. There is less literature on how MSM experience private healthcare facilities compared to public healthcare facilities; however, participants noticed positive interpersonal interactions with healthcare workers, who were described as friendly and attentive. The staff in private facilities were perceived as well-trained in the care and management of homosexual patients, fostering an environment where MSM felt respected and treated as individuals. The findings from a study^12^ indicated that nurses believe that MSM prefer private doctors to public healthcare facilities as care is more personalised and private.

Our study’s findings are in agreement with the suggestion that were made by the participants that there is a need to expand the MSM-friendly and centred health services together with well-trained healthcare providers who do not have judgemental and discriminatory attitudes.^38^ In addition, our study concurs with other suggestions by an earlier study^39^ that there should be an improvement in terms of sensitised care with well-trained healthcare providers, MSM-friendly, inclusive and advocacy for MSM services.

Many participants reported being mislabelled during consultations in public health facilities. Healthcare workers often imposed labels on them, forcing them into the LGBTQI category as gay individuals. Healthcare workers call them with different titles such as Mrs. lady, mam, girl, while some were addressed by their original names. Even though other studies are not entirely showing that MSM are called by the names identified from our study, another study found that MSM are called with different names such as ‘moffie, istabane’.^18^ Our study supports the other findings of other studies that MSM could fear to seek healthcare services because of the labelling or bad experiences from the healthcare providers, for example, an earlier study^35^ stated that most MSM reported fear to seek healthcare services and avoiding such services because of healthcare providers finding out that they are MSM.

The study also uncovered judgemental attitudes from healthcare workers in public facilities towards MSM. Some participants felt unwelcome, with healthcare workers introducing religious beliefs into discussions about their sexuality, which was portrayed as something unacceptable by God. The similar findings regarding religion towards sexual and gender minorities (SGMs) were found in a previous study also.^40^ Our study is in line with an earlier study, which was conducted among the healthcare providers themselves and they acknowledged and portrayed a judgemental attitude towards MSM.^12^

Participants described instances of discrimination and stigmatisation by healthcare workers in public health facilities. They found that healthcare workers conveyed that heterosexual men have a lower risk of contracting sexual illnesses compared to MSM. Furthermore, some healthcare workers did not allow MSM participants to provide input on the services received, suggesting that it was not within their rights as patients. These findings align with earlier studies, which demonstrated that MSM clients faced stigma and discrimination in the healthcare facilities after disclosing their sexual orientation to the healthcare providers.^33,41,42,43^

### Strength and limitations

Several drawbacks to this study have been highlighted. Initially, all the interviews were supposed to take place at the specified clinics, but because of the participants’ personal circumstances and preferences, none of them did. Some of the interviews were carried out in the researcher’s vehicle, whereas others took place in a secluded environment free of noise from people moving around.

The process of finding participants for this study was not easy. The snowball sampling approach was used, which enhanced the recruitment, but several participants were hesitant to provide the researcher with the contact information of people they knew as MSM for fear of damaging their friendships or being accused of breach of commitment for not disclosing their MSM sexualities. However, the sampling method enabled sufficient data until it was saturated.

### Recommendations

Based on the findings in this study, the emergence of this population group is without a doubt inevitable in the cold face of a harsh, hostile and unwelcoming healthcare environment towards this minority group. Healthcare workers and the healthcare system have been portrayed as turning a blind eye and a deaf ear to the call for addressing MSM healthcare needs. Nothing is being done to create a conducive healthcare environment that is welcoming and attractive to MSM, despite the evidence presented in various studies conducted on this phenomenon, which shows that this minority population group is at high risk of being infected with sexually transmitted infections and HIV and of transmitting it to others.

It is in this regard that an inviting and welcoming healthcare environment is recommended for MSM to have equal access to quality healthcare services without being judged, discriminated against or stigmatised because of their sexuality. An MSM-friendly healthcare environment could be created through embedding MSM-targeted healthcare services in existing healthcare programmes in healthcare facilities. Healthcare workers should create an enabling environment for MSM to freely disclose their sexuality and open up about their healthcare issues by demonstrating a non-judgemental, non-discriminative and non-stigmatising attitude towards MSM. Healthcare facilities manager and healthcare workers in particular should take into consideration MSM-specific healthcare needs in their planning and resourcing of healthcare facilities to make them feel included and accommodated in the healthcare system, thus instilling in them a strong sense of belonging. Plans should be put in place to capacitate healthcare workers in healthcare facilities to become culturally competent and skilled in providing healthcare services to MSM. Marketing, advocacy and demand creation for MSM-targeted healthcare services in and out of healthcare facilities should be scaled up through health promotion activities, dialogue and outreach campaigns. The establishment and maintenance of MSM support structures in healthcare facilities could also assist in creating platforms for MSM to raise important issues, which need to be addressed and could provide a much-needed support structure to empower the parents, relatives, and families of MSM.

## Conclusion

In conclusion, the findings of this study have portrayed MSM as entangled, isolated, side-lined, marginalised, and disenfranchised minority population group because of their sexual orientation considering the perceived and experienced difficulties and challenges that they reported going through when accessing healthcare services in public healthcare facilities. The challenges and difficulties that MSM encounter when accessing healthcare services include interpersonal, structural and systematic factors that create barriers and impede MSM from gaining equal access to healthcare services, compared to the heterosexual populations. This underscores an immediate need for tailored approaches to remove these obstacles and promote a more inclusive and equitable healthcare setting for MSM. Unravelling these inequities is critical to improving public health outcomes and for ensuring that health services are framed in the human rights agenda, with universal equitable access.

## References

[1] Bhatt J, Bathija P. Ensuring access to quality health care in vulnerable communities. Acad Med. 2018;93(9):1271–1275. 10.1097/ACM.0000000000002254 29697433 PMC6112847

[2] Commission J. Robert Wood Johnson Foundation initiative on the future of nursing at the Institute of Medicine. Washington, DC: National Academies Press; 2010.

[3] National Department of Health, Republic of South Africa. National sexual & reproductive health and rights policy integrated. 1st ed. Pretoria: National Department of Health Library Cataloguing-in-Publication Data; 2021.

[4] World Health Organization. Quality health services. Geneva: WHO; 2020.

[5] Loue S. Defining men who have sex with men (MSM). Health issues confronting minority men who have sex with men. Springer; 2008, p. 3–37.

[6] Pachauri S, Pachauri A, Mittal K. Men who have sex with men. In: Pachauri S, Pachauri A, Mittal K, editors. Sexual and reproductive health and rights in India: Self-care for universal health coverage. Singapore: Springer; 2022, p. 9–25.

[7] Sileo KM, Baldwin A, Huynh TA, et al. Assessing LGBTQ+ stigma among healthcare professionals: An application of the health stigma and discrimination framework in a qualitative, community-based participatory research study. J Health Psychol. 2022;27(9):2181–2196. 10.1177/13591053211027652 35924592 PMC9642061

[8] Jan S, Manzoor S, Rashid J. Experiences of stigma and discrimination of women living with HIV/AIDS in health-care settings of Kashmir. Indian J Public Health. 2023;67(1):155–158. 10.1089/lgbt.2021.0452 37039222

[9] Ni Z, Shrestha R, Earnshaw VA, et al. Exploring Malaysian physicians’ intention to discriminate against gay, bisexual, and other men who have sex with men patients. LGBT Health. 2023;10(2):169–175. 10.1089/lgbt.2021.0452 36251945 PMC9986015

[10] Van der Elst EM, Gichuru E, Muraguri N, et al. Strengthening healthcare providers’ skills to improve HIV services for MSM in Kenya. AIDS. 2015;29:S237–S240. 10.1097/QAD.0000000000000882 26372492 PMC4706371

[11] Rees SN, Crowe M, Harris S. The lesbian, gay, bisexual and transgender communities’ mental health care needs and experiences of mental health services: An integrative review of qualitative studies. J Psychiatr Ment Health Nurs. 2021;28(4):578–589. 10.1111/jpm.12720 33295065

[12] Seretlo RJ, Mokgatle MM. Practice, attitudes and views of right to access of sexual and reproductive health services by LGBTQI among primary health care nurses in Tshwane. Afr J Prim Health Care Fam Med. 2023;15(1):3790. 10.4102/phcfm.v15i1.3790 36744461 PMC9900286

[13] Philbin MM, Hirsch JS, Wilson PA, Ly AT, Giang LM, Parker RG. Structural barriers to HIV prevention among men who have sex with men (MSM) in Vietnam: Diversity, stigma, and healthcare access. PLoS One. 2018;13(4):e0195000. 10.1371/journal.pone.0195000 29614104 PMC5882136

[14] Helms JD, Tran A, Carnes N, Nehl EJ. Barriers to HIV related services among men who have sex with men (MSM) in rural Georgia. J Georgia Public Health Assoc. 2022;8(3):118–129. 10.20429/jgpha.2022.080315

[15] Ramadhani HO, Ndembi N, Nowak RG, et al. Individual and network factors associated with HIV care continuum outcomes among Nigerian MSM accessing health care services. J Acquir Immune Defic Syndr. 2018;79(1):e7–e16. 10.1097/QAI.0000000000001756 29781881 PMC6092228

[16] Magesa DJ, Leshabari M. Perceived barriers to access available health services among men who have sex with men in Dar es Salaam, Tanzania. Tanzania J Health Res. 2017;19(4):1–9.

[17] Rispel LC, Metcalf CA, Cloete A, Moorman J, Reddy V. You become afraid to tell them that you are gay: health service utilization by men who have sex with men in South African cities. J Public Health Policy. 2011;32:S137–S151. 10.1057/jphp.2011.29 21730987

[18] Duby Z, Nkosi B, Scheibe A, Brown B, Bekker L-G. ‘Scared of going to the clinic’: Contextualising healthcare access for men who have sex with men, female sex workers and people who use drugs in two South African cities. S Afr J HIV Med. 2018;19(1):1–8. 10.4102/sajhivmed.v19i1.701 PMC5843994 29568645

[19] Seretlo RJ, Mokgatle MM, editors. Primary healthcare nurse’s barriers and facilitators to providing sexual and reproductive healthcare services of LGBTQI individuals: A qualitative study. Healthcare. Basel: MDPI; 2022. 10.3390/healthcare10112208 PMC9690274 36360550

[20] Moagi MM, van Der Wath AE, Jiyane PM, Rikhotso RS. Mental health challenges of lesbian, gay, bisexual and transgender people: An integrated literature review. Health SA Gesondheid. 2021;26(1):1–10. 10.4102/hsag.v26i0.1487 PMC7876969 33604059

[21] Sefolosha A, Van Wyk N, Van der Wath A. Reframing personal and professional values: A substantive theory of facilitating lesbian, gay, bisexual, transgender and intersex youth-inclusive primary health care by nurses. J Homosex. 2021;68(8):1298–1319. 10.1080/00918369.2019.1696106 31799891

[22] Mphela AP, Seretlo RJ, Mokwena K, Mokgatle MM. A self-reported study: Health and mental health status among MSM in a district of North-West province. Int J Innov Res Sci Stud. 2024;7(1):9–17. 10.53894/ijirss.v7i1.2398

[23] Rebe K, Lewis D, Myer L, et al. A cross sectional analysis of gonococcal and chlamydial infections among men-who-have-sex-with-men in Cape Town, South Africa. PLoS One. 2015;10(9):e0138315. 10.1371/journal.pone.0138315 26418464 PMC4587970

[24] Stenger MR, Baral S, Stahlman S, Wohlfeiler D, Barton JE, Peterman T. As through a glass, darkly: The future of sexually transmissible infections among gay, bisexual and other men who have sex with men. Sex Health. 2016;14(1):18–27. 10.1071/SH16104 PMC5334461 27585033

[25] Ma W, Chen Z, Niu S. Advances and challenges in sexually transmitted infections prevention among men who have sex with men in Asia. Curr Opin Infect Dis. 2023;36(1):26–34. 10.1097/QCO.0000000000000892 36480294 PMC9794152

[26] Malefo MA, Ayo-Yusuf O, Mokgatle MM. Risk factors for sexually transmitted infections among men who have sex with men. Afr J Prim Health Care Fam Med. 2023;15(1):4080. 10.4102/phcfm.v15i1.4080 37916720 PMC10623483

[27] Le Roux M, Ngwenya IK, Nemarude AL, De Villiers BE, Mathebula M, Nchabeleng M. Sexually transmitted infections and sexual behaviour among men having sex with men from Tshwane, South Africa. Int J STD AIDS. 2023;34(3):183–190. 10.1177/09564624221146673 36542494

[28] Wray TB, Pantalone DW, Kahler CW, Monti PM, Mayer KH. The role of discrimination in alcohol-related problems in samples of heavy drinking HIV-negative and positive men who have sex with men (MSM). Drug Alcohol Depend. 2016;166:226–234. 10.1016/j.drugalcdep.2016.07.017 27481457 PMC4983543

[29] Garcia A, Rowe C, Turner C, Santos G-M. Correlates of alcohol-using network size among men who have sex with men in San Francisco, CA. Am J Men’s Health. 2021;15(2):15579883211007005. 10.1177/15579883211007005 33899602 PMC8076769

[30] Müller A. Scrambling for access: Availability, accessibility, acceptability and quality of healthcare for lesbian, gay, bisexual and transgender people in South Africa. BMC Int Health Hum Rights. 2017;17:1–10. 10.1186/s12914-017-0124-4 28558693 PMC5450393

[31] Braun V, Clarke V. Using thematic analysis in psychology. Qual Res Psychol. 2006;3(2):77–101. 10.1191/1478088706qp063oa

[32] Isano S, Yohannes T, Igihozo G, Ndatinya GI, Wong R. A qualitative study to explore the healthcare-seeking experiences of men who have sex with men (MSM) and transgender women (TGW) in Rwanda. BMC Health Serv Res. 2023;23(1):291. 10.1186/s12913-023-09286-x 36978054 PMC10045920

[33] Magesa DJ, Mtui LJ, Abdul M, et al. Barriers to men who have sex with men attending HIV related health services in Dar es Salaam, Tanzania. Tanzania J Health Res. 2014;16(2):118–126. 10.4314/thrb.v16i2.8 26875306

[34] Kushwaha S, Lalani Y, Maina G, et al. “But the moment they find out that you are MSM…”: A qualitative investigation of HIV prevention experiences among men who have sex with men (MSM) in Ghana’s health care system. BMC Public Health. 2017;17:1–18. 10.1186/s12889-017-4799-1 28974257 PMC5627492

[35] Mbeda C, Ogendo A, Lando R, et al. Healthcare-related stigma among men who have sex with men and transgender women in sub-Saharan Africa participating in HIV Prevention Trials Network (HPTN) 075 study. AIDS Care. 2020;32(8):1052–1060. 10.1080/09540121.2020.1776824 32500722 PMC7368806

[36] Bolderston A, Ralph S. Improving the health care experiences of lesbian, gay, bisexual and transgender patients. Radiography. 2016;22(3):e207–e211. 10.1016/j.radi.2016.04.011

[37] Salerno JP, Turpin R, Howard D, Dyer T, Aparicio EM, Boekeloo BO. Health care experiences of black transgender women and men who have sex with men: A qualitative study. J Assoc Nurs AIDS Care. 2020;31(4):466–475. 10.1097/JNC.0000000000000111 31274661

[38] Kapanda L, Jumbe V, Izugbara C, Muula AS. Healthcare providers’ attitudes towards care for men who have sex with men (MSM) in Malawi. BMC Health Serv Res. 2019;19:1–9. 10.1186/s12913-019-4104-3 31101107 PMC6525370

[39] Mwaniki SW, Kaberia PM, Mugo PM, Palanee-Phillips T. “We must help them despite who they are…”: Healthcare providers’ attitudes and perspectives on care for young gay, bisexual and other men who have sex with men in Nairobi, Kenya. BMC Health Serv Res. 2023;23(1):1055. 10.1186/s12913-023-10026-4 37789339 PMC10546658

[40] Joudeh L, Harris OO, Johnstone E, Heavner-Sullivan S, Propst SK. “Little red flags”: barriers to accessing health care as a sexual or gender minority individual in the rural Southern United States—A qualitative intersectional approach. J Assoc Nurs AIDS Care. 2021;32(4):467–480. 10.1097/JNC.0000000000000271 PMC8238829 33935190

[41] Risher K, Adams D, Sithole B, et al. Sexual stigma and discrimination as barriers to seeking appropriate healthcare among men who have sex with men in Swaziland. J Int AIDS Soc. 2013;16:18715. 10.7448/IAS.16.3.18715 24242263 PMC3833105

[42] Gamariel F, Isaakidis P, Tarquino IAP, et al. Access to health services for men who have sex with men and transgender women in Beira, Mozambique: A qualitative study. PLoS One. 2020;15(1):e0228307. 10.1371/journal.pone.0228307 31999760 PMC6992190

[43] Moyo I, Macherera M, Mavhandu-Mudzusi AH. The lived experiences of men who have sex with men when accessing HIV care services in Zimbabwe. Health SA Gesondheid (Online). 2021;26:1–8. 10.4102/hsag.v26i0.1462 PMC8111641 34007471

